# Failure Detection in Deep Neural Networks for Medical Imaging

**DOI:** 10.3389/fmedt.2022.919046

**Published:** 2022-07-22

**Authors:** Sabeen Ahmed, Dimah Dera, Saud Ul Hassan, Nidhal Bouaynaya, Ghulam Rasool

**Affiliations:** ^1^Department of Electrical and Computer Engineering, Rowan University, Glassboro, NJ, United States; ^2^University of Texas Rio Grande Valley, Brownsville, TX, United States; ^3^AMD Inc., Austin, TX, United States; ^4^Machine Learning Department, Moffitt Cancer Center, Tampa, FL, United States

**Keywords:** failure detection, robustness, trustworthiness, adversarial attacks, Bayesian deep neural networks, self-assessment, reliability, natural noise

## Abstract

Deep neural networks (DNNs) have started to find their role in the modern healthcare system. DNNs are being developed for diagnosis, prognosis, treatment planning, and outcome prediction for various diseases. With the increasing number of applications of DNNs in modern healthcare, their trustworthiness and reliability are becoming increasingly important. An essential aspect of trustworthiness is detecting the performance degradation and failure of deployed DNNs in medical settings. The softmax output values produced by DNNs are not a calibrated measure of model confidence. Softmax probability numbers are generally higher than the actual model confidence. The model confidence-accuracy gap further increases for wrong predictions and noisy inputs. We employ recently proposed Bayesian deep neural networks (BDNNs) to learn uncertainty in the model parameters. These models simultaneously output the predictions and a measure of confidence in the predictions. By testing these models under various noisy conditions, we show that the (learned) predictive confidence is well calibrated. We use these reliable confidence values for monitoring performance degradation and failure detection in DNNs. We propose two different failure detection methods. In the first method, we define a fixed threshold value based on the behavior of the predictive confidence with changing signal-to-noise ratio (SNR) of the test dataset. The second method learns the threshold value with a neural network. The proposed failure detection mechanisms seamlessly abstain from making decisions when the confidence of the BDNN is below the defined threshold and hold the decision for manual review. Resultantly, the accuracy of the models improves on the unseen test samples. We tested our proposed approach on three medical imaging datasets: PathMNIST, DermaMNIST, and OrganAMNIST, under different levels and types of noise. An increase in the noise of the test images increases the number of abstained samples. BDNNs are inherently robust and show more than 10% accuracy improvement with the proposed failure detection methods. The increased number of abstained samples or an abrupt increase in the predictive variance indicates model performance degradation or possible failure. Our work has the potential to improve the trustworthiness of DNNs and enhance user confidence in the model predictions.

## 1. Introduction

Artificial Intelligence (AI) and Machine Learning (ML) are among the pivotal technologies modernizing clinical practices, medical diagnostics, and healthcare. Every day, new ML and deep neural networks (DNNs) applications are being explored in various clinical application areas, including medical imaging, clinical text and language processing, and clinical decision support systems. When considered for deployment in clinical settings, trustworthiness, reliability, and robustness are among the primary challenges modern ML techniques face, including DNNs.

Modern DNNs have achieved high performance in various areas, including computer vision, natural language processing, and reinforcement learning. Several techniques based on DNNs, including convolutional neural networks (CNNs), recurrent neural networks (RNNs), Transformers, and Graph Neural Networks (GNNs), have revolutionized various areas of human endeavor. Modern DNNs seem to perform very well on specific tasks; however, they are susceptible to out-of-distribution changes in the input data and natural as well as adversarial noise. DNNs are fragile and perform poorly when confronted with noisy inputs. These brittle models may fail without warning after deployment in real-world mission-critical settings.

The primary contributing reasons for the possible failure of DNNs after deployment include; (1) aleatoric uncertainty, resulting from intrinsic noise in the observed data, such as noise from sensors producing noisy data, and (2) epistemic uncertainty, representing the uncertainty in model parameters due to a lack of available training data (1). In a clinical setting, examples of these uncertainties include various types of day-to-day changes in machine settings, changes in recorded data due to hardware/software upgrades, noise related to human errors, etc. Adversarial attacks are another possible source of model failure. Adversarial samples consist of imperceptible perturbations in the input data that fool the DNNs into making wrong predictions (2). Modern DNNs should perform robustly in the face of various types of natural noise and adversarial attacks.

DNNs should be able to assess their own performance degradation and warn the users about their possible failure. For DNNs employed in modern healthcare systems, the inability to identify performance degradation may have fatal repercussions (3–7). Performance monitoring after deployment requires an accurate self-assessment mechanism since there is no ground truth available to track model accuracy anymore. Self-assessment refers to monitoring model's confidence (or alternately uncertainty) in its decisions by itself. Thus, leveraging accurate uncertainty quantification may enable reliable failure detection that will enhance trustworthiness and reliability.

Various techniques exist in the literature for measuring a model's confidence in its decision. For example, in the case of a multi-class classification task, the output of the softmax function, also referred to as softmax probability, available at the output of DNNs is used as an uncertainty estimate. However, softmax output values do not represent a DNN's confidence in its decision. These uncalibrated softmax probability numbers generally represent overconfidence and are not appropriate for DNN failure detection. We propose to use a Bayesian DNN (BDNN) technique, referred to as Variational Density Propagation (VDP) that defines the model parameters as random variables and outputs predictive distribution instead of softmax probability. VDP-based BDNNs propagate the mean and the variance of the model parameters through all the layers of the DNN and output predictive distribution. Assuming a Gaussian distribution, the model simultaneously produces two outputs; the decision (represented by the mean of the distribution) and the associated uncertainty (quantified by the variance of the distribution). In our framework, the uncertainty is learned during training instead of estimating *post-hoc* after training. We also verify the reliability of our uncertainty estimates by performing extensive experiments and observing the trends of the predictive variance under different levels of Gaussian noise and various types of adversarial attacks.

We propose two failure detection methods based on the observed patterns from experiments on the predictive variance. Both methods are based on defining a threshold on the predictive variance available at the output of the BDNN. During inference (after deployment), if the predictive variance of the input sample is below the threshold, the output is passed on for downstream processing. Otherwise, if the predictive variance is above the threshold, the BDNN abstains and outputs “no decision.”

The first proposed method is based on manually selecting the threshold and is referred to as the *fixed threshold*. The manual selection of the threshold variance value is based on the behavior of the predictive variance and model performance under various noise levels. In the second method, we *learn* a threshold using a neural network. The neural network is trained using clean and noisy images to learn the threshold variance value. The confident predictions are retained based on each image's learned threshold, and the learned model abstains from under-confident decisions, i.e., when the predictive variance is above the threshold.

We tested our proposed method on three different medical imaging datasets, (1) PathMNIST, (2) DermaMNIST, and (3) OrganAMNIST. We evaluated the performance of the former method using three different fixed threshold values. We trained two different models with different hyperparameters for each dataset for the learned threshold. In our experiments, after applying the threshold, we note considerable improvement in the accuracy, especially at high noise levels. However, the number of abstained cases (“no decision”) also increased. The average post-threshold accuracy improvement is 24.2, 16.0, and 16.4% when tested under Gaussian noise for PathMNIST, DermaMNIST, and OrganAMNIST datasets, respectively. The average number of abstained decisions is 53.0, 51.7, and 51.2% for PathMNIST, DermaMNIST, and OrganAMNIST datasets under Gaussian noise. Similar results are observed for the adversarial attacks.

In our experiments, we observe a trade-off between improved accuracy after applying the threshold and the number of abstained decisions. As a general trend, we observe that as accuracy improvement increases, the number of abstained samples also increases. The choice of the type of threshold (learned vs. fixed) and subsequent parameter selection is dependent upon the dataset and application. The proposed failure detection methods considerably enhance model performance, provide robust means of model failure detection, and help users establish trust in the AI/ML models.

The paper is structured as follows. The Section 2 provides details of the framework that forms the basis of our proposed work, along with datasets and details about the experiments. Next, in Section 3, we present our results for three medical imaging datasets. In Section 4, we provide a detailed overview of existing methodologies in the literature and an analysis of our experimental observations. We conclude by summarizing our work and outlining directions for future work in Section 5.

## 2. Materials and Methods

This section starts with the description of the mathematical fundamentals of the Bayesian methodology used in our work to estimate uncertainty. Next, we provide details of our two methods proposed for failure detection in deep neural networks. The last part of the section comprehensively presents the setup for our experiments, including model architectures, medical datasets, compute resources, frameworks, noise types and levels employed in model testing/inference.

### 2.1. Mathematical Preliminaries

#### 2.1.1. Bayesian Deep Learning

We consider model parameters (weights and biases) as random variables and define a probability distribution function over these parameters known as the *prior* distribution function. After observing the data, Bayes' rule is used to find the *posterior* probability distribution function. Given *N* samples of the training data, $D={\left\{X^{\left(i\right)},y^{\left(i\right)}\right\}}_{i=1}^{N}$, and the prior distribution defined over the model parameters θ~*p*(θ), we can apply Bayes' theorem. However, the posterior probability distribution function *p*(θ|*D*) is generally intractable due to a large number of model parameters and the functional form of the neural network, which does not lend itself to exact integration (8). Variational inference approximates the true posterior *p*(θ|*D*) with a parameterized variational distribution *q*_ϕ_(θ) that is easy to estimate. The optimal parameters ϕ^*^ of the variational probability distribution function are estimated by minimizing the Kullback-Leibler (KL) divergence between the approximate and the true posterior distribution functions. The resulting relationship, referred to as the evidence lower bound (ELBO), can be maximized to find the optimal parameters ϕ^*^ of the posterior distribution function (9). We can also construct a loss function from ELBO that can be minimized by the gradient descent algorithm.


(1) $$\begin{array}{l} ℒ=-{\text{E}}_{q_{\phi }(\theta )}[\text{log }p(D|\theta )]+\text{KL}(q_{\phi }(\theta )||p(\theta )). \end{array}$$

#### 2.1.2. Variational Density Propagation (VDP)

The variational density propagation framework, proposed by Dera et al. (10), propagates the first two moments of the variational distribution *q*_ϕ_(θ) through the layers of the model, including all linear and nonlinear transformations. Consequently, all the operations performed on model parameters and data are considered operations on random variables, which include (1) multiplication of random variables with a constant, (2) multiplication of two random variables, and (3) non-linear transformations operating on random variables. We start by defining model parameters as Gaussian random variables. However, the outputs of various (non-)linear operations performed at different layers of the neural network may not follow the Gaussian distribution (11). In such cases, we assume that the first two moments can still estimate and represent the underlying probability distribution function (10). VDP model output consists of the mean and the variance of the predictive distribution *p*(**ỹ** **|*X*, θ**), without performing any sampling operation as required in other methods (8, 12). The mean of the predictive distribution function is the model's decision, and the variance-covariance matrix represents the associated uncertainty with the decision.

##### Model Description

We consider our neural network to have *C* convolutional layers, *L* fully-connected layers, activation functions, max-pooling layers, batch-normalization layers, skip connections, and softmax function. The network's parameters are represented by $\theta =\left\{{\left\{W^{\left(k_{c}\right)}\right\}}_{k_{c}=1}^{K_{c}},{\left\{W^{\left(l\right)}\right\}}_{l=1}^{L}\right\}$, where ${\left\{W^{\left(k_{c}\right)}\right\}}_{k_{c}=1}^{K_{c}}$ is the set of *K*_*c*_ kernels in the *c*th convolutional layer, and ${\left\{W^{\left(l\right)}\right\}}_{l=1}^{L}\}$ is the set of weights in *L* fully connected layers. The kernels are defined under the tensor normal distributions of order 3, $W\sim {\mathrm{TN}}_{n_{1},n_{2},n_{3}}\left(M,V\right)$, where M is the mean tensor, and V is the covariance tensor. Assuming the neural network input and the model parameters are uncorrelated with each other, the mean and covariance after each operation performed in the layers of the neural network can be described by relationships in the following paragraphs (10, 13–15).

**Convolutional Operation—First Layer**: Considering the input *X* as a constant we have,


(2) $$\begin{array}{l} z^{\left(k_{1}\right)}=X*W^{\left(k_{1}\right)}=\tilde{X}\times \text{vec}(W^{\left(k_{1}\right)}), \end{array}$$

where $\tilde{X}$ is formed by arranging vectorized sub-matrices of *X* (having the same dimensions as the kernel) into rows, $W^{\left(k_{1}\right)}$ is the *k*th kernel of the first convolution layer (*c* = 1), and * denotes the convolution operation. The output of the convolutional layer is given by,


(3) $$\begin{array}{l} {\text{z}}^{\left(k_{s}\right)}\sim N\left(\mu_{{\text{z}}^{\left(k_{1}\right)}}=\tilde{X}{\text{m}}^{\left(k_{1}\right)},\Sigma_{{\text{z}}^{\left(k_{1}\right)}}=\tilde{X}\Sigma^{\left(k_{1}\right)}{\tilde{X}}^{T}\right), \end{array}$$

where ${\text{m}}^{\left(k_{1}\right)}=\text{vec}\left(M^{\left(k_{1}\right)}\right)$ and $\Sigma^{\left(k_{1}\right)}=\text{vec}\left(V^{\left(k_{1}\right)}\right)$.

**Non-linear Activation Function**: The mean and covariance after a non-linear activation function, *f*, are derived using the first-order Taylor series approximation (11). Let ${\text{a}}^{\left(k_{c}\right)}=\text{f}\left({\text{z}}^{\left(k_{c}\right)}\right)$, then the mean and covariance of ${\text{a}}^{\left(k_{c}\right)}$ are derived as,


(4) $$\begin{array}{l} \mu_{{\text{a}}^{\left(k_{c}\right)}}\approx \text{f}\left(\mu_{{\text{z}}^{\left(k_{c}\right)}}\right),\text{and}\Sigma_{{\text{a}}^{\left(k_{c}\right)}}\approx \Sigma_{{\text{z}}^{\left(k_{c}\right)}}⊙\left(\nabla \text{f}\left(\mu_{{\text{z}}^{\left(k_{c}\right)}}\right)\nabla \text{f}{\left(\mu_{{\text{z}}^{\left(k_{c}\right)}}\right)}^{T}\right), \end{array}$$

where ∇ is the first order derivative with respect to ${\text{z}}^{\left(k_{c}\right)}$ and ⊙ is the Hadamard product.

**Max-Pooling Operation**: The mean and covariance at the output of the max-pooling operation are given by,


(5) $$\begin{array}{l} \mu_{p^{\left(k_{c}\right)}}=\text{pool}(\mu_{a^{\left(k_{c}\right)}}),\text{ and }\Sigma_{p^{\left(k_{c}\right)}}=\text{co-pool}(\Sigma_{a^{\left(k_{c}\right)}}), \end{array}$$

where pool represents the max-pooling operation, and co-pool represents down-sampling the covariance matrix to keep the rows and columns corresponding to the pooled elements of the mean.

**Flattening Operation**: The output *P* of the max-pooling layer is vectorized to form $\text{b}\;=\;\left[{\text{p}}^{\left(1\right)T},\ldots ,{\text{p}}^{\left(K_{c}\right)T}\right]$. The mean and covariance at the output of the flattening operation are given by,


(6) $$\begin{array}{l} \mu_{\text{b}}=\left[\begin{array}{c} \mu_{{\text{p}}^{\left(1\right)}}\\ \vdots \\ \mu_{{\text{p}}^{\left(K_{c}\right)}} \end{array}\right],\Sigma_{\text{b}}=\left[\begin{array}{ccc} \Sigma_{{\text{p}}^{\left(1\right)}} & \cdots & 0\\ \vdots & \ddots & \vdots \\ 0 & \cdots & \Sigma_{{\text{p}}^{\left(K_{c}\right)}} \end{array}\right]. \end{array}$$

**Fully-Connected Layer**: Let ${\text{w}}_{i}\sim N\left({\text{m}}_{i},\Sigma_{i}\right)$ be the *i*^th^ weight vector of the fully-connected layer and *i* = 1, …, S (S are the number of classes or equivalently the number of output nodes). Let **d** be the output of the fully connected-layer, then *d*_*i*_ = **bw**_*i*_ is the product of two random vectors.


(7) $$\begin{array}{l} \mu_{d_{i}}=m_{i}^{T}\mu_{b},\\ \Sigma_{d}=\{\begin{array}{ll} \text{tr}\left(\Sigma_{i}\Sigma_{b}\right)+m_{i}^{T}\Sigma_{b}m_{j}+\mu_{b}^{T}\Sigma_{i}\mu_{b}, & i=j\\ m_{i}^{T}\Sigma_{b}m_{j}, & i\neq j, \end{array} \end{array}$$

where, *i, j* = 1, …, S.

**Softmax Function**: Let *g* be the softmax function, then **ỹ** = *g*(**d**). Using the first order Taylor series approximation, the mean and the covariance of **ỹ** are derived as,


(8) $$\begin{array}{l} \mu_{\tilde{y}}\approx \text{g}(\mu_{d}),\text{ and }\Sigma_{\tilde{y}}\approx J_{\text{g}}\Sigma_{d}J_{\text{g}}^{T}. \end{array}$$

where, **J** is the Jacobian of the softmax function with respect to **d**.

**Convolution Operation—Intermediate Layers**: In this layer, the previous layer's input is considered as a Gaussian random variable. Like the first convolution layer, sub-matrices of the input are formed to have the same dimensions as the kernels. Multiplication of the vectorized sub-matrices and the vectorized kernels are a product of two uncorrelated random vectors. Therefore, the resulting mean and covariance are given by Equations (7).

**Batch Normalization Layer**:


(9) $$\begin{array}{l} \mu_{y_{i}}^{BN}=\frac{\gamma }{\sqrt{\sigma_{ℬ}^{2}+\epsilon }}⊙(\mu_{x_{i}}-\;l\mu_{ℬ})+\beta , \end{array}$$

(10) $$\begin{array}{ll} \Sigma_{{\text{y}}_{i}}^{BN} & =\text{Diag}\left(\frac{\gamma }{\sqrt{\sigma_{B}^{2}+\epsilon }}\right)\Sigma_{{\text{x}}_{i}}\text{Diag}\left(\frac{\gamma }{\sqrt{\sigma_{B}^{2}+\epsilon }}\right), \end{array}$$

where, **x**_*i*_ are the input vectors, B is a mini-batch of the input vectors, $\mu_{B}\text{and}\sigma_{B}^{2}$ are the sample mean and variance over the mini-batch, γ and β are hyper-parameters for scaling and shifting the input, ϵ is a constant for numerical stability, and Diag(*x*) is a diagonal matrix with diagonal entries of the vector **x**.

**Residual Connection**: Residual or skip connections perform identity mapping to skip a few layers in a model (16). The skip connection relationship can be derived as,


(11) $$\begin{array}{l} {\text{x}}_{t+1}={\text{x}}_{t}+F\left({\text{x}}_{t}\right)\text{ and }\mu_{{\text{x}}_{t+1}}\approx \mu_{{\text{x}}_{t}}+F\left(\mu_{{\text{x}}_{t}}\right);\Sigma_{{\text{x}}_{t+1}}\approx \text{J}\Sigma_{{\text{x}}_{t}}{\text{J}}^{T}, \end{array}$$

where, **x**_*t*_and**x**_*t*+1_ are the input and output vectors of the *t*th block in the model, F is the non-linear residual function, and **J** is Jacobian of **x**_*t*+1_ with respect to **x**_*t*_.

**The Loss Function**: We assume that the covariance matrix for the initial variational distribution is diagonal, *N* independently and identically distributed data points are given, and *M* Monte Carlo samples can approximate the expectation by a summation. The loss function given in Equation (1), can be implemented using the following two relations.


(12) $$\begin{array}{l} \text{Expected Log-likelihood:}\\ E_{q_{\phi }(\Omega )}\{\log p(\tilde{y}|X,\theta )\}\approx -\frac{NH}{2}\log(2\pi )\\ -\frac{1}{M}\sum_{m=1}^{M}[\frac{N}{2}\log(|\Sigma_{\tilde{y}}|)\text{​}\\ +\text{​}\frac{1}{2}\sum_{i\text{​}=\text{​}1}^{N}{\left(y^{\left(i\right)}\text{​}-\text{​}\mu_{\tilde{y}}^{\left(m\right)}\right)}^{T}{\left(\Sigma_{\tilde{y}}^{\left(m\right)}\right)}^{-1}(y^{\left(i\right)}\text{​}-\text{​}\mu_{\tilde{y}}^{\left(m\right)})]. \end{array}$$

(13) $$\begin{array}{l} \text{Regularization:}\\ \text{KL}\left[q_{\phi }\left(\theta \right)∥p\left(\theta \right)\right]=\overset{C}{\underset{c=1}{\sum }}\overset{K_{c}}{\underset{k_{c}=1}{\sum }}\text{KL}\left[q_{\phi }\left(W^{\left(k_{c}\right)}\right)∥p\left(W^{\left(k_{c}\right)}\right)\right]\\ +\overset{L}{\underset{l=1}{\sum }}\text{KL}\left[q_{\phi }\left(W^{\left(l\right)}\right)∥p\left(W^{\left(l\right)}\right)\right]. \end{array}$$

### 2.2. Failure Detection Methods

In our settings, a model is said to fail when its performance drops below a certain predefined level. However, after deployment, we do not have access to the ground truth labels and cannot assess the model's performance. We propose using VDP-based Bayesian deep neural networks (BDNNs) that output a measure of confidence and the decision. We can use this confidence or uncertainty information to assess the model's performance and ascertain its failure. We propose two methods for defining the threshold on the uncertainty (variance of the predictive distribution). At test time or during deployment, if the predictive variance is more than the threshold, the model will abstain and output “no decision.”

#### 2.2.1. Fixed Threshold

**Figure 1:**
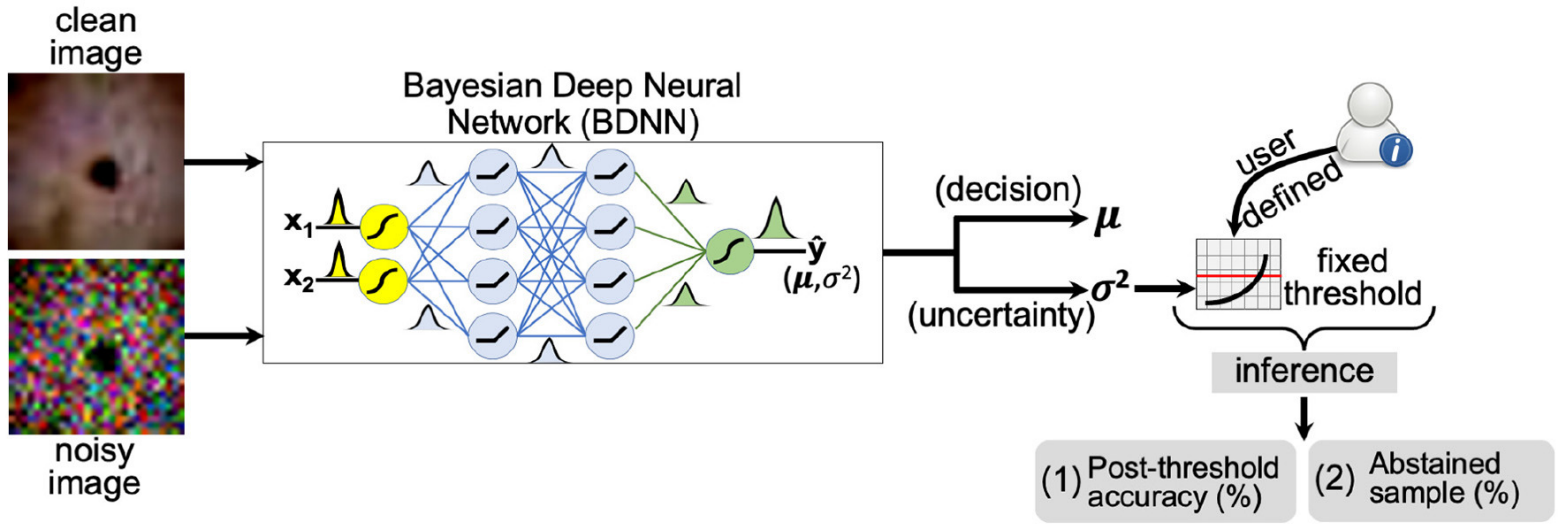
A schematic layout of the fixed threshold method is presented. The user defines the threshold based on predictive variance trend with varying noise levels (SNR) of the test dataset. If the predictive variance is above the defined threshold value, the model abstains or outputs “no decision” at the inference time.

We establish the reliability of the uncertainty (or the predictive variance) estimated by the BDNN. We observe the behavior of the predictive variance and corresponding accuracy under different types and levels of natural and adversarial noise. The pattern of three different predictive variance values is tracked, corresponding to the correct decisions, incorrect decisions, and combined (correct + incorrect). We manually select a fixed threshold value based on the observed patterns of these three types of predictive variances and the model's accuracy. A schematic layout of the proposed approach is presented in Figure 1. We experimented with three different threshold values for comparison and quantified the effect of different threshold values on the model's post-threshold accuracy and the number of abstained samples. The first threshold value is selected based on the median predictive variance corresponding to the correct decisions at the minimum tested noise level. This threshold value corresponds to the highest accuracy. The next two threshold values are selected using the combined and correct variance trends. Four different bins of test samples are used to test the selected threshold levels. These bins include clean test images and images with low, medium, and high Gaussian noise. The post-threshold accuracy and percentage of abstained samples are recorded for each threshold value and for the four bins (no, low, medium, high noise) of test images.

#### 2.2.2. Learning Threshold Using Neural Networks

**Figure 2:**
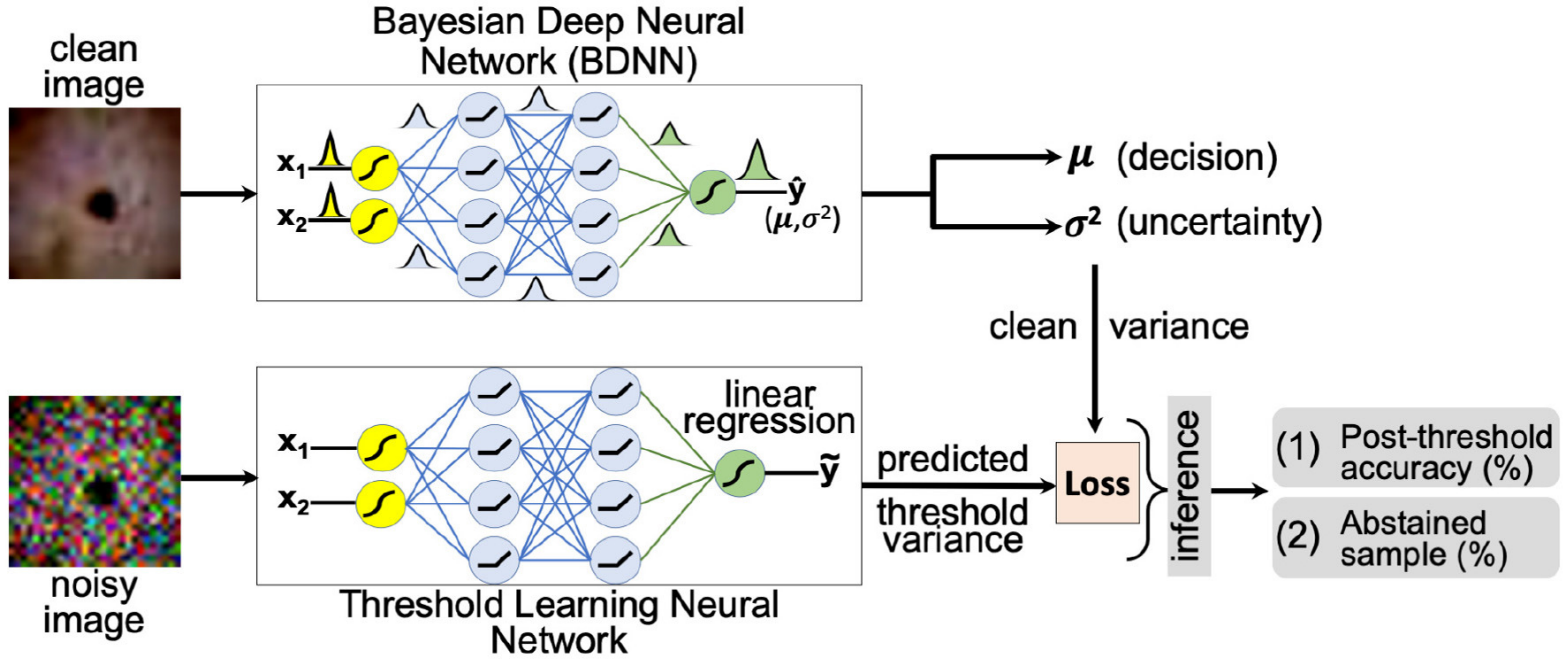
A schematic layout of the threshold learning method is presented. The threshold variance is learned using a neural network. At the inference time, the model abstains or outputs “no decision” if the predictive variance is above the learned threshold value. The threshold is learned using both the clean and the noisy training dataset.

The second method attempts to learn the threshold value for the predictive variance using a neural network. A schematic layout of the proposed approach is presented in Figure 2. We aim to learn a threshold value that is numerically close to the predictive variance of the clean images produced by the BDNN model. The *input* to the threshold learning model consists of clean and noisy training dataset samples. The noisy training samples are created using different levels of additive Gaussian noise. The *target output* for the neural network consists of predictive variance values as estimated by the BDNN for corresponding clean training samples. We formulate this as a regression problem and use the mean-square error or mean absolute error loss function. We report the two different sets of results based on two different hyperparameter settings. We use different model architectures depending on the dataset. Generally, a combination of convolutional and fully-connected layers or only fully-connected layers are used. We use four different test bins; clean testing images and testing images with additive Gaussian noise of low, medium, and high level. Then we tested the learned threshold model against adversarial attacks, despite the threshold learning model being never exposed to adversarial attacks. We created separate test data bins for each type and level of adversarial attack. We recorded the post-threshold accuracy and number of abstained test samples (in percentage) for each test bin.

### 2.3. Experimental Setup

**Figure 3:**
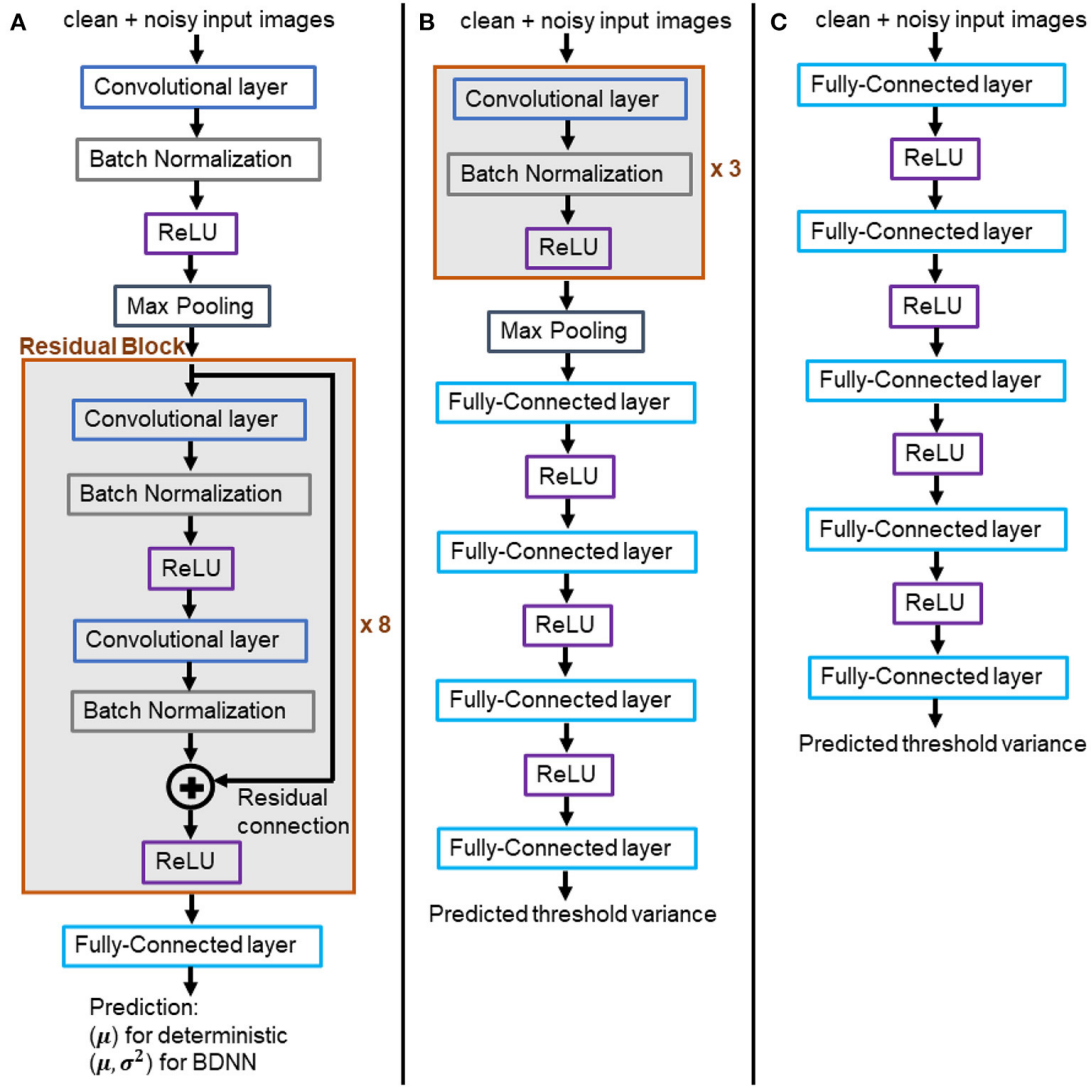
**(A)** Architecture of the trained BDNN models for uncertainty quantification. Deterministic models share the same architecture. **(B,C)** Models for learning the threshold variance. **(B)** Learned threshold model for PathMNIST dataset. **(C)** Learned threshold model for DermaMNIST and OrganAMNIST datasets.

We trained two flavors of Resnet18 models, (1) deterministic and (2) VDP-based BDNNs. The former models output softmax probability values, which serve as the decision (the largest value) and are also considered model confidence, albeit uncalibrated. VDP-based BDNNs produce two separate outputs simultaneously, the decision and the uncertainty associated with the decision. Figure 3A presents the architecture of Resnet18 models used in our study. Deterministic and BDNN models share the same architecture. We train both sets of deterministic and BDNN Resnet18 models using original clean training datasets. The training, validation, testing, and hyperparameter optimization are done using web-based visualization tool, *Weights and Biases* (17). All experiments are performed at test time on the trained models using the test datasets. We report the mean of uncalibrated softmax probability in deterministic models. The reported output variance of BDNN models is using the median values for the tested samples.

Two different architectures have been used to learn the threshold variance for the output variances that the BDNN models generate for each decision, as presented in Figures 3B,C. The model illustrated in Figure 3B is used to learn the threshold variance for the PathMNIST dataset, whereas the model in Figure 3C is used for DermaMNIST and OrganAMNIST datasets. Details on the compute resources, frameworks, and software packages used in our experiments are given in Table 1. Code base for this work is available at: https://github.com/Beemd/Failure-Detection_MedMNIST.

**Table 1:** Compute resources, frameworks, and packages.

**Environment/conditions**	**Package name**	**Version**
Compute resources	NVIDIA TITAN RTX GPU,	–
	48 GB GPU Memory	
Operating systems	Ubuntu	20.04.3
	Windows	10
Programming languages	Python	3.8.10
Deep learning framework	Pytorch	1.8.1
	Pytorch-lightning	1.2.5
	Torchvision	0.9.1
Adversarial library	Torchattacks	3.2.2
Miscellaneous	Scikit-image	0.18.1
	Scikit-learn	0.24.2
	Pandas	1.2.4
	Numpy	1.20.2

#### 2.3.1. Analysis Using Gaussian Noise

We used different levels of Gaussian noise to corrupt the test dataset. We quantify the noise level added in each test data sample using the signal-to-noise ratio or SNR. At high noise levels, the SNR is low and *vice versa*. SNR is calculated using:


$$\begin{array}{l} \text{SNR (dB)}=10\log_{10}\frac{X_{\text{signal}}}{X_{\text{noise}}}, \end{array}$$

where *X*_signal_ is the clean test data sample and *X*_noise_ is the noise. Table 2 presents the Gaussian noise levels and their corresponding SNR values for the three datasets used in our experiments. We define four test bins [“no noise,” “low noise,” “medium noise,” “high noise”]. Each bin uses all samples from the test datasets.

**Table 2:** Gaussian noise levels and corresponding SNR values used in our experiments.

**Noise level**	**SNR (dB)**		
	**PathMNIST**	**DermaMNIST**	**OrganAMNIST**
Minimum tested	56.40	56.12	54.69
Low	26.89	26.60	20.74
Medium	22.52	19.31	13.27
High	16.71	14.79	6.27
Maximum tested	13.42	10.75	2.59

#### 2.3.2. Analysis Using Adversarial Attacks

Adversarial attacks introduce imperceptible perturbations in the input using optimization techniques, rendering the attacks unnoticed by human operators (2). These malicious inputs are frequently used to test the robustness of deep neural networks (18). We used three different types of adversarial attacks, the Fast Gradient Sign Method (FGSM) (19), Projected Gradient Descent (PGD) (20), and Carlini and Wagner (CW) (21). Further, we used four different levels of FGSM attacks. For FGSM attacks, the ϵ values were set to [0.001, 0.005, 0.01, 0.1], [0.0005, 0.002, 0.004, 0.008], and [0.005, 0.02, 0.04, 1.2] for PathMNIST, DermaMNIST, and OrganAMNIST, respectively. In PGD, we set ϵ = [0.005, 0.004, 0.02] for PathMNIST, DermaMNIST, OrganAMNIST, respectively. We set α = 2/255 and used 50 steps for the optimization. The confidence values were set to *c* = [0.05, 0.01, 0.95] for CW attacks on PathMNIST, DermaMNIST, and OrganAMNIST, respectively. We used κ = 0, steps = 100, and *lr* = 0.01. We defined a test bin for each type and level of attack with 400 samples selected randomly from the test datasets.

#### 2.3.3. Datasets

We used a subset of a large, curated biomedical images dataset, referred to as *MedMNIST* (22). We used PathMNIST, DermaMNIST, and OrganAMNIST for classification tasks in our experiments. A summary of the datasets is given in Table 3. PathMNIST dataset is built on colorectal cancer (CRC) histology slides used in a study on survival prediction (23). The study provides a dataset based on 86 hematoxylin-eosin (HE)-stained slides of human cancer tissue from the National Center for Tumor diseases (NCT) (24) biobank and the University Medical Center Mannheim (UMM) pathology archive to form 100, 000 (NCT-CRC-HE-100K) non-overlapping image patches with nine classes: adipose tissue, background, debris, lymphocytes, mucus, smooth muscle, normal colon mucosa, cancer-associated stroma, and CRC epithelium. The images in NCT-CRC-HE-100 K are split into training and validation sets with a ratio of 9:1. The test dataset of 7, 180 image patches is built using 25 HE slides of human CRC tissue from the “Darmkrebs: Chancen der Verhütung durch Screening” (DACHS) study (25) in the NCT biobank. The source images of 3 × 224 × 224 are resized to 3 × 28 × 28.

**Table 3:** Summary of the datasets.

	**No. of classes**	**Image size**	**Image channels**	**Dataset format**	**No. of samples**		
					**Training set**	**Validation set**	**Test set**
PathMNIST	9	28 ×28	3	Numpy Serialization Files (npz)	89,996	10,004	7,180
DermaMNIST	7		3		7,007	1,003	2,005
OrganAMNIST	11		1		34,581	6,491	17,778

DermaMNIST dataset is built on HAM10000 (26, 27), a large collection of dermatoscopic images of pigmented melanocytic and common non-melanocytic skin lesions. Non-pigmented lesions are not part of this dataset. HAM10000 comprises 10, 015 images collected from multiple sources. These images were filtered, pathologic diagnoses was unified, standardized, and a final manual quality review was run to form seven generic classes: akiec [Actinic Keratoses (Solar Keratoses) and Intraepithelial Carcinoma (Bowen's disease)], bcc (Basal cell carcinoma), bkl (Benign keratosis), df (Dermatofibroma), nv (Melanocytic nevi), mel (Melanoma), and vasc (Vascular skin lesions). The images are split into training, validation and test sets with a ratio of 7:1:2. The source images of 3 × 600 × 450 are resized to 3 × 28 × 28.

**Figure 4:**
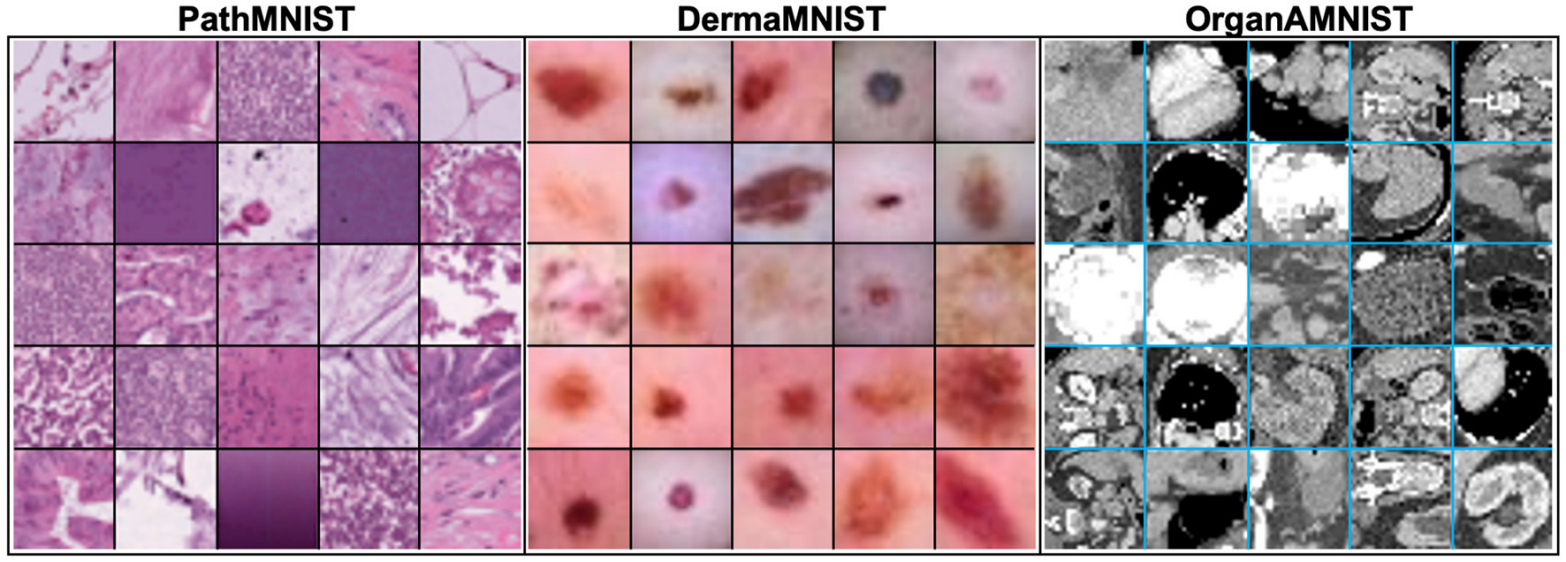
Sample images of input data from PathMNIST, DermaMNIST, OrganAMNIST datasets used in our experiments.

OrganAMNIST dataset is built on contrast-enhanced abdominal computed tomography (CT) images from Liver Tumor Segmentation Benchmark (LiTS) (28). LiTS consists of 201 scans collected from different clinical sites around the world. Organ labels are obtained using bounding-box annotations of 11 body organs from another study (29): heart, left lung, right lung, liver, spleen, pancreas, left kidney, right kidney, bladder, left femoral head, and right femoral head. The study used segmentation masks provided by LiTS to compute the bounding-box for the liver. The bounding-box for the other organs is annotated and verified by radiologists. The source training set of 131 CT scans is split into training and validation sets with 115 and 16 CT scans, respectively. The source test set of 70 CT scans is used as the test set. Voxel intensities of the 3D scans are transformed from Hounsfield-Unit (HU) into gray-scale. OrganAMNIST dataset is the 2D images cropped from the center slices of the 3D bounding-boxes in axial view (plane). The source images are resized to 1 × 28 × 28. The sample images of each dataset are shown in Figure 4.

## 3. Results

In this section, we present the results of our experiments that include deterministic and Bayesian Resnet18 models trained and evaluated using PathMNIST, DermaMNIST, and OrganAMNIST datasets.

### 3.1. The Accuracy-Probability Gap

**Figure 5:**
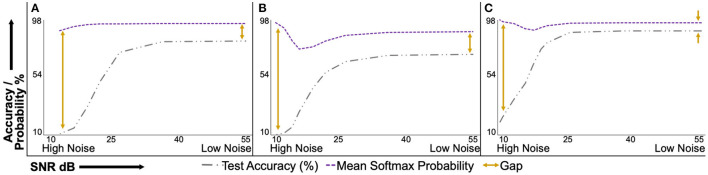
Uncalibrated mean softmax probability and corresponding test accuracy of deterministic models under varying signal-to-noise ratio (SNR). The plots are conveniently interpreted if read from right to left on the horizontal axis. The mean softmax probability is higher than the model accuracy at the minimum tested noise level (right side of the horizontal axis). As the noise level increases, the accuracy-probability gap continues to widen. This behavior shows the overconfidence of deterministic models, especially at higher noise levels. **(A)**, PathMNIST; **(B)**, DermaMNIST; and **(C)**, OrganAMNIST.

Figure 5 presents the test accuracy and corresponding uncalibrated softmax probability values of the deterministic models under varying natural noise levels. Figures 5A–C present results for PathMNIST, DermaMNIST, and OrganAMNIST datasets, respectively. It is convenient to interpret sub-figures from right to left on the horizontal axis, that is, going from low noise (or high SNR) to high noise (or low SNR). The accuracy-probability gap for all three datasets at the minimum tested noise level is 13.39, 17.24, and 6.24%. The gap increases to 79.97, 87.64, and 78.48% at the maximum tested noise level for respective datasets. The accuracy-probability gap is a qualitative measure of the lack of confidence calibration in deterministic deep neural networks.

### 3.2. Predictive Variance Under Noise

**Figure 6:**
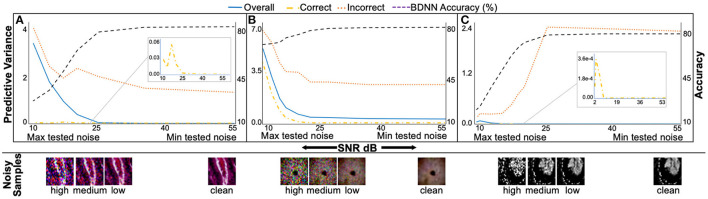
The median predictive variance (left vertical axis) and BDNNs' test accuracy values (right vertical axis) are plotted against varying levels of Gaussian noise added to the test datasets. In addition to the test accuracy in each sub-figure, three predictive variance curves, refer to the correct, incorrect, and combined (correct + incorrect) predictions. Inset in sub-figures shows the zoomed-in correct predictive variance trend. Sample test images from different noise levels are also presented. **(A)**, PathMNIST; **(B)**, DermaMNIST, and **(C)** OrganAMNIST.

Figure 6 presents median predictive variance and test accuracy plots with varying levels of additive Gaussian noise for BDNNs. The noise levels are quantified using SNR. Figures 6A–C present results for PathMNIST, DermaMNIST, and OrganAMNIST datasets, respectively. In addition to test accuracy in each sub-figure, three separate curves for the predictive variance are presented. The variance curves illustrate median predictive variance values for the (1) correct decisions, (2) incorrect decisions, and (3) combined (correct + incorrect). Predictive variance refers to the left vertical axis in all sub-figures. Each sub-figure has the BDNN's test accuracy (right vertical axis) evaluated at various noise levels.

It is evident from Figure 6 that the BDNN predictive variance values increase with increasing noise for all datasets. The range of predictive variance for the PathMNIST dataset is ~10^4^, 10^2^, and 2.7 for combined, correct, and incorrect decisions, respectively. These values are 10, 10^2^, 3.9 for DermaMNIST dataset, and 10^7^, 10^6^, 10 for OrganAMNIST dataset.

Table 4 shows the performance comparison of BDNN and deterministic models under three different types of adversarial attacks, PGD, FGSM, and CW, for all three datasets. Table 4 also presents predictive variance values of correct, incorrect, and combined (correct + incorrect) decisions. In general, the predictive variance increases with the addition of noise, which serves as a surrogate for the out-of-distribution testing in our settings. Such an increase in the variance is the basis of our proposed failure detection mechanisms.

**Table 4:** Performance comparison of BDNN and deterministic models under adversarial attacks.

**Dataset**	**Attack**	**Attack**	**Test accuracy (**%**)**		**Median predictive variance**		
	**type**	**level**	**Deterministic**	**BDNN**	**Combined**	**Correct**	**Incorrect**
PathMNIST	PGD	0.005	30.00	45.25	0.129091	0.121537	0.13909
	FGSM	0.001	75.75	81.00	7.82E-03	1.24E-03	1.58E+00
		0.005	35.00	48.75	1.62E-01	4.68E-02	2.93E-01
		0.01	16.25	21.00	2.82E-02	5.00E-04	6.27E-02
		0.1	9.25	13.75	5.15E-02	1.74E-03	2.42E-01
	CW	c = 0.05	0.25	43.75	1.139096	1.77E-07	6.736739
DermaMNIST	PGD	0.004	35.25	63.50	5.54E-01	3.39E-01	1.10E+00
	FGSM	0.0005	63.75	73.00	3.87E-01	6.02E-02	2.74E+00
		0.002	47.25	69.00	4.34E-01	9.33E-02	1.92E+00
		0.004	32.25	64.50	5.04E-01	2.24E-01	1.33E+00
		0.008	16.50	55.25	7.88E-01	6.15E-01	9.00E-01
	CW	c = 0.01	0.25	64.25	3.36E-01	7.87E-03	4.95E+00
OrganAMNIST	PGD	0.02	43.00	63.00	5.84E-05	1.86E-06	3.28E-03
	FGSM	0.005	83.00	83.25	1.14E-07	2.08E-09	1.23E+00
		0.02	58.50	66.00	4.52E-05	1.06E-07	3.35E-02
		0.04	36.50	47.00	2.11E-05	3.38E-08	1.17E-03
		1.20	15.25	9.75	8.49E-03	6.23E-08	1.57E-02
	CW	c = 0.95	1.00	69.25	3.76E-09	2.42E-11	21.39

### 3.3. Detection of Failure in BDNNs

We propose two mechanisms of failure detection in BDNNs using the predictive variance information: (1) a fixed threshold on the variance and (2) a neural network based learnable threshold. Tables 5–7 present failure detection results for PathMNIST, DermaMNIST, and OrganAMNIST datasets, respectively. Trained BDNNs were tested under three different levels of Gaussian noise for failure detection using both methods, fixed and learnable. The presented results include the threshold values (for the fixed threshold method), accuracy before applying the threshold on the variance (whether fixed or learned), percentage of abstained test samples (because their associated predictive variance was over the threshold), and the updated accuracy excluding the abstained samples.

**Table 5:** Detection of failure in BDNNs—PathMNIST dataset tested under various levels of Gaussian noise.

**Noise**	**Accuracy prior to threshold (%)**	**Threshold**	**Threshold value**	**Abstained test samples (%)**	**Accuracy post threshold (%)**
None	84.25	Fixed	0.0008	50.26	96.78
			0.002	47.09	96.63
			0.03	36.38	95.69
		Learned	ver-1	20.29	91.75
			ver-2	23.43	91.05
Low	79.79	Fixed	0.0008	62.02	97.47
			0.002	58.80	97.13
			0.03	48.13	95.92
		Learned	ver-1	32.05	91.54
			ver-2	22.92	91.11
Medium	65.39	Fixed	0.0008	74.18	97.36
			0.002	71.95	96.57
			0.03	63.61	93.88
		Learned	ver-1	50.89	86.70
			ver-2	47.86	84.35
High	35.04	Fixed	0.0008	86.42	93.23
			0.002	84.35	92.08
			0.03	77.62	84.26
		Learned	ver-1	75.63	81.26
			ver-2	67.72	68.72

*The terms ver-1 and ver-2 correspond to different hyperparameter settings*.

**Table 6:** Detection of failure in BDNNs—DermaMNIST dataset tested under various levels of Gaussian noise.

**Noise**	**Accuracy prior to threshold (%)**	**Threshold**	**Threshold value**	**Abstained test samples (%)**	**Accuracy post threshold (%)**
None	73.27	Fixed	0.08	60.90	93.88
			0.20	55.66	92.35
			1.00	35.61	87.37
		Learned	ver-1	32.82	85.23
			ver-2	43.54	89.40
Low	72.37	Fixed	0.08	60.65	92.97
			0.20	57.06	90.59
			1.00	37.26	86.41
		Learned	ver-1	33.92	84.75
			ver-2	44.99	88.67
Medium	68.33	Fixed	0.08	64.79	90.23
			0.20	62.20	89.22
			1.00	46.14	83.98
		Learned	ver-1	39.55	82.43
			ver-2	51.37	85.95
High	63.39	Fixed	0.08	79.00	77.67
			0.20	73.32	78.50
			1.00	58.85	78.06
		Learned	ver-1	52.97	75.08
			ver-2	66.53	79.43

*The terms ver-1 and ver-2 correspond to different hyperparameter settings*.

**Table 7:** Detection of failure in BDNNs—OrganAMNIST dataset tested under various levels of Gaussian noise.

**Noise**	**Accuracy prior to threshold (%)**	**Threshold**	**Threshold value**	**Abstained test samples (%)**	**Accuracy post threshold (%)**
None	90.22	Fixed	2.00E-08	45.96	99.72
			1.00E-05	31.96	99.17
			2.00E-03	22.04	98.07
		Learned	ver-1	33.23	99.21
			ver-2	44.39	99.66
Low	89.13	Fixed	2.00E-08	48.91	99.59
			1.00E-05	34.47	98.82
			2.00E-03	24.06	97.68
		Learned	ver-1	35.77	98.98
			ver-2	47.13	99.51
Medium	78.05	Fixed	2.00E-08	62.75	96.62
			1.00E-05	47.32	93.99
			2.00E-03	34.58	91.01
		Learned	ver-1	48.37	94.30
			ver-2	60.96	96.63
High	42.47	Fixed	2.00E-08	89.82	79.67
			1.00E-05	75.78	69.34
			2.00E-03	58.56	60.54
		Learned	ver-1	77.07	71.03
			ver-2	88.62	79.69

*The terms ver-1 and ver-2 correspond to different hyperparameter settings*.

Tables 8–10 present failure detection results under three different adversarial attacks for PathMNIST, DermaMNIST, and OrganAMNIST datasets, respectively. Each table shows results for PGD, CW, and four different levels of the FGSM attacks. The tables provide test accuracy before the failure detection, the level and types (fixed vs. learned) of the threshold for failure detection, the percentage of abstained test samples with variance beyond the threshold, and post-threshold test accuracy.

**Table 8:** Detection of failure in BDNNs—PathMNIST dataset tested under various types of adversarial attacks.

**Attack**	**Attack level**	**Accuracy prior to threshold (%)**	**Threshold type**	**Threshold**	**Abstained test samples (%)**	**Accuracy post threshold (%)**
FGSM	0.001	81.00	Fixed	0.0008	49.25	96.55
				0.002	48.00	96.15
				0.03	38.25	93.52
			Learned	ver-1	24.00	88.82
				ver-2	27.25	88.66
	0.005	48.75	Fixed	0.0008	69.00	66.94
				0.002	67.25	64.89
				0.03	57.75	59.17
			Learned	ver-1	40.25	50.63
				ver-2	43.00	49.56
	0.01	21.00	Fixed	0.0008	56.75	31.21
				0.002	55.25	30.17
				0.03	45.00	25.91
			Learned	ver-1	28.75	22.81
				ver-2	34.75	18.77
	0.1	13.75	Fixed	0.0008	56.75	22.54
				0.002	56.00	22.73
				0.03	49.00	24.51
			Learned	ver-1	43.75	18.67
				ver-2	38.25	21.05
CW	c = 0.05	43.75	Fixed	0.0008	62.25	98.68
				0.002	57.75	98.22
				0.03	56.00	97.16
			Learned	ver-1	50.50	88.38
				ver-2	50.25	86.93
PGD	0.005	45.25	Fixed	0.0008	75.50	67.35
				0.002	72.50	63.64
				0.03	59.25	50.31
			Learned	ver-1	38.50	43.90
				ver-2	42.00	42.67

*The terms ver-1 and ver-2 correspond to different hyperparameter settings*.

**Table 9:** Detection of failure in BDNNs—DermaMNIST dataset tested under various types of adversarial attacks.

**Attack**	**Attack level**	**Accuracy prior to threshold (%)**	**Threshold type**	**Threshold**	**Abstained test samples (%)**	**Accuracy post threshold (%)**
FGSM	0.0005	73.00	Fixed	0.08	60.00	93.75
				0.2	57.00	91.28
				1	37.00	86.51
			Learned	ver-1	28.50	82.52
				ver-2	50.25	87.44
	0.002	69.00	Fixed	0.08	61.00	86.54
				0.2	57.00	84.88
				1	39.75	80.50
			Learned	ver-1	29.75	74.02
				ver-2	53.25	81.82
	0.004	64.50	Fixed	0.01	64.25	81.12
				0.2	58.50	77.11
				1	42.00	71.98
			Learned	ver-1	35.00	67.69
				ver-2	57.50	73.53
	0.008	55.25	Fixed	0.08	69.25	58.54
				0.2	62.50	58.67
				1	46.00	57.41
			Learned	ver-1	42.50	52.17
				ver-2	65.25	57.55
CW	c = 0.01	64.25	Fixed	0.08	59.25	95.09
				0.2	56.75	93.64
				1	37.50	87.60
			Learned	ver-1	30.50	82.01
				ver-2	49.00	88.73
PGD	0.004	63.50	Fixed	0.08	63.00	77.70
				0.2	58.25	74.85
				1	41.75	70.39
			Learned	ver-1	34.75	64.37
				ver-2	57.25	70.18

*The terms ver-1 and ver-2 correspond to different hyperparameter settings*.

**Table 10:** Detection of failure in BDNNs—OrganAMNIST dataset tested under various types of adversarial attacks.

**Attack**	**Attack level**	**Accuracy prior to threshold (%)**	**Threshold type**	**Threshold**	**Abstained test samples (%)**	**Accuracy post threshold (%)**
FGSM	0.005	83.25	Fixed	2.00E-08	46.25	99.53
				1.00E-05	36.75	97.63
				2.00E-03	31.75	94.87
			Learned	ver-1	43.50	99.12
				ver-2	52.75	100.00
	0.02	66.00	Fixed	2.00E-08	60.00	88.13
				1.00E-05	47.25	81.52
				2.00E-03	42.00	79.74
			Learned	ver-1	55.00	84.44
				ver-2	65.00	92.14
	0.04	47.00	Fixed	2.00E-08	57.50	62.35
				1.00E-05	46.50	57.94
				2.00E-03	41.75	54.94
			Learned	ver-1	53.50	62.37
				ver-2	65.25	68.35
	1.2	9.75	Fixed	2.00E-08	73.00	19.44
				1.00E-05	62.50	14.67
				2.00E-03	57.00	13.95
			Learned	ver-1	69.00	16.94
				ver-2	79.75	23.46
CW	c = 0.95	69.25	Fixed	2.00E-08	39.00	99.59
				1.00E-05	33.25	99.63
				2.00E-03	30.75	98.92
			Learned	ver-1	36.50	99.61
				ver-2	45.25	100.00
PGD	0.02	63.00	Fixed	2.00E-08	61.50	79.87
				1.00E-05	48.25	73.91
				2.00E-03	43.50	72.57
			Learned	ver-1	57.75	79.29
				ver-2	70.75	86.32

*The terms ver-1 and ver-2 correspond to different hyperparameter settings*.

## 4. Discussion

Trustworthiness is the primary challenge for DNNs in healthcare applications. The availability of uncertainty information or a measure of confidence in the output decision of DNNs may help users (like medical practitioners) establish confidence in these models. We build on our prior work in density propagation BDNNs (4, 10, 13, 14, 30). These BDNNs output predictive variance along with the decision or prediction, which serves as a calibrated measure of confidence or equivalently uncertainty. We proposed and evaluated two different failure detection methods. The first one is based on the fixed threshold applied to the predictive variance. In the second case, a neural network is used to learn the threshold value. In both cases, the BDNN model is considered to fail when the predictive variance is above the threshold, whether fixed or learned. In such cases, the model abstains from making any decision, and the produced output is “no decision.” By abstaining from uncertain decisions, the proposed threshold methods substantially improve the performance of BDNNs (based on the accuracy of confident decisions), especially when the model faces corrupt or out-of-distribution inputs. We use Gaussian noise and adversarial attacks to corrupt the input images at test time.

### 4.1. Existing Approaches to Failure Detection and Uncertainty Estimation

Different techniques have been proposed in the literature to estimate uncertainty in model predictions and detect when the model is failing. We broadly segment these techniques into *non-Bayesian* and *Bayesian* categories.

#### 4.1.1. Non-Bayesian Methods

Many different approaches have been proposed in the literature. Generally, the output of the softmax function, where available, is used as the model confidence (31). However, the softmax output is not a calibrated measure of model confidence (15). The raw softmax probabilities overestimate model confidence for the correct as well as incorrect predictions (15). We categorize the existing non-Bayesian methods into three main areas; (1) calibration techniques, (2) *ad-hoc* methods, and (3) ensemble techniques, as explained in the following paragraphs.

The calibration techniques help models adjust the softmax output values such that these values represent the model confidence. Among calibration techniques, *temperature scaling* uses a scalar parameter to re-scale logit scores (15). Temperature scaling with small perturbation in input data improves the detection of out-of-distribution samples (32). *Label smoothing* is another calibration technique that diffuses the one-hot encoded labels using a small probability value for incorrect classes. There are various methods for label smoothing, including a data augmentation strategy called *mixup* (33, 34). Mixup employs *vicinal risk minimization*, which uses additional data samples and labels from the original dataset. Moon et al. (35) proposed training DNNs with a different loss function, named *correctness ranking loss*. The correctness ranking loss regularizes the class probabilities such that these values are ordinal rankings based on the true model confidence (35). The *Deep Gamblers* technique is based on the portfolio theory and uses a loss function designed for selective classification problems using the doubling rate of gambling (36). It transforms the m-class classification problem into m+1 classes, with the additional class representing whether the model abstains from making a decision due to low confidence. Calibration techniques are computationally efficient. However, the model outputs decisions and represents the associated confidence in those decisions using the same softmax output values. Calibration techniques are generally not accurate and (except for correctness ranking loss) do not differentiate between the confidence values of correct and incorrect decisions.

*Ad-hoc* methods generally extend the model architecture to predict uncertainty and detect out-of-distribution inputs (37, 38). DeVries and Taylor (37) applied sigmoid function to the logits from the main classification model to estimate confidence as *c*∈[0, 1] for out-of-distribution detection. Corbiere et al. (38) proposed *true class probability* based on feature maps extracted from the main model during training. *SelectiveNet* uses a modified architecture with three heads (prediction, selection, and auxiliary) and a special loss function to optimize classification and rejection simultaneously (39). Although the uncertainty learned by extended model architecture produces acceptable results, these techniques are not suitable for failure detection. These techniques require modification in the model architecture, which may alter model performance. *Ad-hoc* techniques also do not capture the uncertainty introduced by the additional components added to the model architecture.

Ensemble techniques combine the output of multiple models to improve predictive performance. The probability distribution of the point predictions of individual models can be used for uncertainty estimation (40–43). This technique has been extended for out-of-distribution detection (44, 45). Ensemble techniques produce reliable confidence estimates; however, these techniques are computationally expensive. *Trust score* is another non-Bayesian technique for estimating model confidence (46). Trust score is estimated by the degree of agreement between the model and a modified nearest-neighbor classifier. High trust scores correlate to higher precision in the correct classification of samples and can be used to measure certainty (46). Trust score is appropriate for small datasets only as finding the nearest neighbors in large datasets is computationally challenging.

#### 4.1.2. Bayesian Methods

Bayesian methods use Bayes' theorem to calculate the posterior distribution function, which can be used to find the predictive distribution for new samples (47). Some Bayesian methods use variational inference to estimate the posterior distribution, while others use various sampling techniques (47). In variational inference, we propose an easy-to-estimate parameterized posterior distribution and later estimate its parameters using gradient descent during model training (10, 47). Recently, a Bayesian fully-connected deep neural network with the name of *Bayes by Backprop* was proposed by Blundell et al. (8). Bayes by Backprop defines a fully-factorized Gaussian distribution over the model weights and uses variational inference to find its first two moments (8). At the test time, Monte Carlo samples from the learned posterior distribution provide an estimate of the uncertainty in the output decisions. Later on, Shridhar et al. (48) extended Bayes by Backprop from fully-connected neural networks to CNNs. In general, Bayes by Backprop technique is computationally expensive because the number of unknown parameters to be estimated is doubled.

Monte-Carlo dropout, proposed by Gal and Ghahramani, is a Bayesian approximation technique (12). The method uses dropout operation at the inference time for the estimation of uncertainty in DNNs (12). Monte-Carlo dropout is computationally efficient as compared to Bayes by Backprop. However, both Bayes by Backprop and Monte-Carlo dropout use sampling for the estimation of uncertainty in the output decision. Both techniques do not propagate uncertainty (the second moment or the variance of the variational distribution) through the network layers, that is, the model does not learn the uncertainty during training.

Dirichlet prior network (DPN) is based on the Bayesian framework but uses a Dirichlet distribution as a prior over the predictive categorical distribution (49, 50). DPN is shown to be similar to DNNs for classification using softmax output, but the difference is in the loss function. DPN performs well for out-of-distribution detection. However, for misclassification detection, DPN relies on maximum softmax probability, which is calibrated.

Our proposed failure detection mechanism is based on our recently introduced Bayesian technique that uses variational inference (10, 13, 14, 30). This technique, referred to as Variational Density Propagation (VDP), propagates the first two moments of the variational distribution through all mathematical operations, linear and non-linear, in the model layers. VDP-based models produce two outputs, that is, the first two moments of the variational distribution. The output mean is the model's decision, and the variance is the uncertainty in the decision. VDP introduces only a few additional parameters and is computationally not expensive (10, 13, 14).

### 4.2. The Accuracy-Probability Gap

Figures 5A–C present test accuracy and softmax probability values at different noise levels for the deterministic models trained for PathMNIST, DermaMNIST, and OrganAMNIST, respectively. Ideally, for a calibrated model, the accuracy-probability gap should be zero. In such cases, the softmax probability values represent the output decision and the model confidence. In Figure 5, we observe that the accuracy-probability gap always exists for all tested datasets and increases with the noise level. This gap highlights that the uncalibrated softmax probability output values are an overconfident representation of the model confidence (15). The raw or uncalibrated softmax output probability values are not a reliable representation of model uncertainty and should not be used to detect out-of-distribution samples or model failure.

### 4.3. Predictive Variance Under Noise

The test accuracy and corresponding predictive variance values for BDNNs for a range of noise levels are presented in Figure 6. We observe that the test accuracy drops with the increasing noise level, whereas the median predictive variance values increase. Such behavior establishes the coherence between the model's accuracy and confidence in its decisions. The predictive variance of BDNNs captures the uncertainty introduced by different noise levels. The increase in predictive variance for correct decisions shows that the model's confidence in its correct decisions decreases as the noise increases. We also observe that the predictive variance of the incorrect decisions is higher than the variance value corresponding to the correct decisions at any given SNR. The consistent gap between the correct and incorrect predictive variance values indicates that the model is relatively unsure about its wrong decisions compared to the correct decisions made at the same SNR level. The observed gap between the predictive variance values for the correct and incorrect decisions and the increase in the predictive variance with increasing noise establishes the predictive variance as a reliable metric for failure detection.

The clean (noise-free) test accuracy values for the deterministic and BDNN models are [82.95, 84.25%], respectively, for PathMNIST, [73.67, 73.27%] for DermaMNIST, and [90.12, 90.22%] for OrganAMNIST datasets. In Figures 5, 6, moving from the minimum to the maximum tested noise levels, we observe that the drop in accuracy of BDNNs is less than the deterministic models. The least drop in accuracy is for DermaMNIST BDNN, which decreased from 73.27 → 61.89% whereas its deterministic counterpart dropped from 73.67 → 11.07% in accuracy. PathMNIST BDNN accuracy drops from 84.25 → 27.16% compared with 82.95 → 10.85% for the deterministic model. The accuracy drop for OrganAMNIST BDNN is from 90.22 → 22.87% which is almost similar to its deterministic counterpart, 90.12 → 20.05%. This performance comparison between BDNNs and deterministic models shows that generally, BDNNs are more robust, as already established in our previous work (10, 13, 14, 30). In other experiments we observe a similar accuracy trend between BDNN and deterministic models for the *speckle* additive noise.

Table 4 presents the performance comparison of BDNN and deterministic models under three different adversarial attacks. We note that the variance values corresponding to incorrect decisions are higher than those corresponding to the correct decisions for all types of attacks. This behavior of predictive variance shows that the BDNN model is relatively more confident about its correct decisions than its incorrect decisions under adversarial attacks. The variance values for combined, correct, and incorrect decisions under all types and levels of attack are higher than the variance values for the clean test dataset. The larger variance values for adversarially attacked samples demonstrate that BDNN is relatively more uncertain when under attacks. These observed patterns form the basis for the failure detection methods proposed in our current work. Table 4 also shows that under increasing levels of FGSM attack, the variance values do not show a consistent increasing or decreasing pattern. This inconsistent behavior is unlike the trend observed for the Gaussian noise. We attribute the behavior of variance under different levels of FGSM attacks to the nature of the optimization problem that the FGSM attacks use to fool the model. Generally, all adversarial attacks use various optimization techniques to find the minimum distortion in the input that will fool the model into changing its decision while remaining imperceptible to the human user. The adversarial attacks find the optimized distortion at each attack level, resulting in inconsistent predictive variance values. Each type of attack uses a different technique; hence the predictive variance values and the difference between the correct and incorrect predictive variance values for each attack varies. In our experiments, we also observed that for stronger PGD attacks (e.g., ϵ = 0.008) for the PathMNIST dataset, the deterministic model fails with the accuracy dropping to 0.25%. In contrast, the accuracy of BDNN for the same level of adversarial attack is 42.75%. However, the predictive variance for incorrect decisions for BDNN is lower than the correct decisions, i.e., 0.44 vs. 0.65.

In Table 4, we also note that the performance of BDNN is better than its deterministic counterpart under all types and levels of adversarial attacks. This performance trend shows that BDNN models are robust to adversarial attacks as already demonstrated in our previous work (10, 13, 14, 30).

### 4.4. Detection of Failure in BDNNs

The failure of a machine learning model can be defined using its accuracy. However, after the deployment of a model, there are no ground truth labels and the accuracy cannot be calculated. Different flavors of BDNNs (e.g., VDP) can be used which output decision as well as some measure of uncertainty in the decision, that is, the variance of the predictive distribution. Using this variance information, we have developed two methods for the failure detection that estimate a failure threshold, named *fixed* and *learnable*. After deployment, if the predictive variance for an input is above the defined threshold value, the model is considered to have failed in providing a reliable and trustworthy decision and the model output is set to “no decision.”

The fixed threshold method uses manually selected predictive variance values. The threshold values are selected by analyzing the variance curves in conjunction with the model accuracy at different noise levels (refer to Figure 6). On the other hand, the learnable threshold method uses a neural network to learn the threshold corresponding to each input sample. The fixed and learned variance values serve as a threshold for segregating decisions made by the model. These predictive variance thresholds block uncertain decisions, improving the remaining decisions' accuracy. The change in the predictive variance values for the correct and incorrect decisions and the behavior of predictive variance under various noise levels establishes the efficacy of the proposed failure detection methods. Both failure detection mechanisms perform better when the overlap between the distributions of correct and incorrect predictive variance is minimum.

### 4.5. Failure Detection Under Gaussian Noise

#### 4.5.1. Fixed Threshold

We note in Tables 5–7 that as the fixed threshold value is increased, the number of abstained samples (“no decision” cases) and the accuracy of confident decisions decreases at the same noise level. When the threshold level is constant and the noise level increases, the number of incorrect decisions increases. Out of these incorrect decisions, the ones whose variance values are above the threshold fall in the “no decision” domain. That means the model abstains and does not produce any output. Consequently, with the increasing noise, the number of abstained samples and improvement in accuracy increases.

The average accuracy improvement for the PathMNIST dataset, after applying the fixed thresholds, are [12.1, 17.1, 30.6, 54.8%] for the cases of [“no noise,” “low noise,” “medium noise,” “high noise”], respectively. The corresponding average abstained samples are [44.6, 56.3, 69.9, 82.8%]. For the DermaMNIST dataset (Table 6), the average accuracy improvement values are [17.9, 17.4, 19.5, 14.7%] and average abstained samples are [50.7, 51.7, 57.7, 70.4%]. For the OrganAMNIST dataset (Table 7), we note that the average accuracy improvement is [8.8, 9.6, 15.8, 27.4%] and the average abstained samples are [33.3, 35.8, 48.2, 74.7%] for all four noise levels, respectively.

#### 4.5.2. Learned Threshold

In Tables 5–7, we observe a relationship between the number of abstained samples and the test accuracy (post-threshold). At two different BDNN training hyperparameter settings, the percentage of abstained samples and the post-threshold accuracy tend to co-vary. That is, a threshold value that provides the largest improvement in the post-threshold test accuracy may also result in a higher percentage of abstained samples. In Table 5, the average accuracy improvement is [7.2, 11.5, 20.1, 40.0%] and average abstained samples are [21.9, 27.5, 49.4, 71.7%] for [“no noise,” “low noise,” “medium noise,” “high noise”], respectively. In Table 6, we observe a similar pattern in accuracy improvement for the DermaMNIST dataset. The average accuracy improvement is [14.0, 14.3, 15.9, 13.9%] and the number of abstained samples are [38.2, 39.5, 45.5, 59.8%]. In Table 7, for the OrganaMNIST dataset, the average accuracy improvement is [9.2, 10.1, 17.4, 32.9%] and the number of abstained samples are [38.8, 41.5, 54.7, 82.8%]. The learned threshold method tends to abstain from fewer samples than the fixed threshold method for PathMNIST and DermaMNIST datasets. Therefore, the increase in post-threshold accuracy is also relatively less. In OrganAMNIST, the learned method abstains from more samples than the fixed threshold and produces higher post-threshold accuracy.

**Figure 7:**
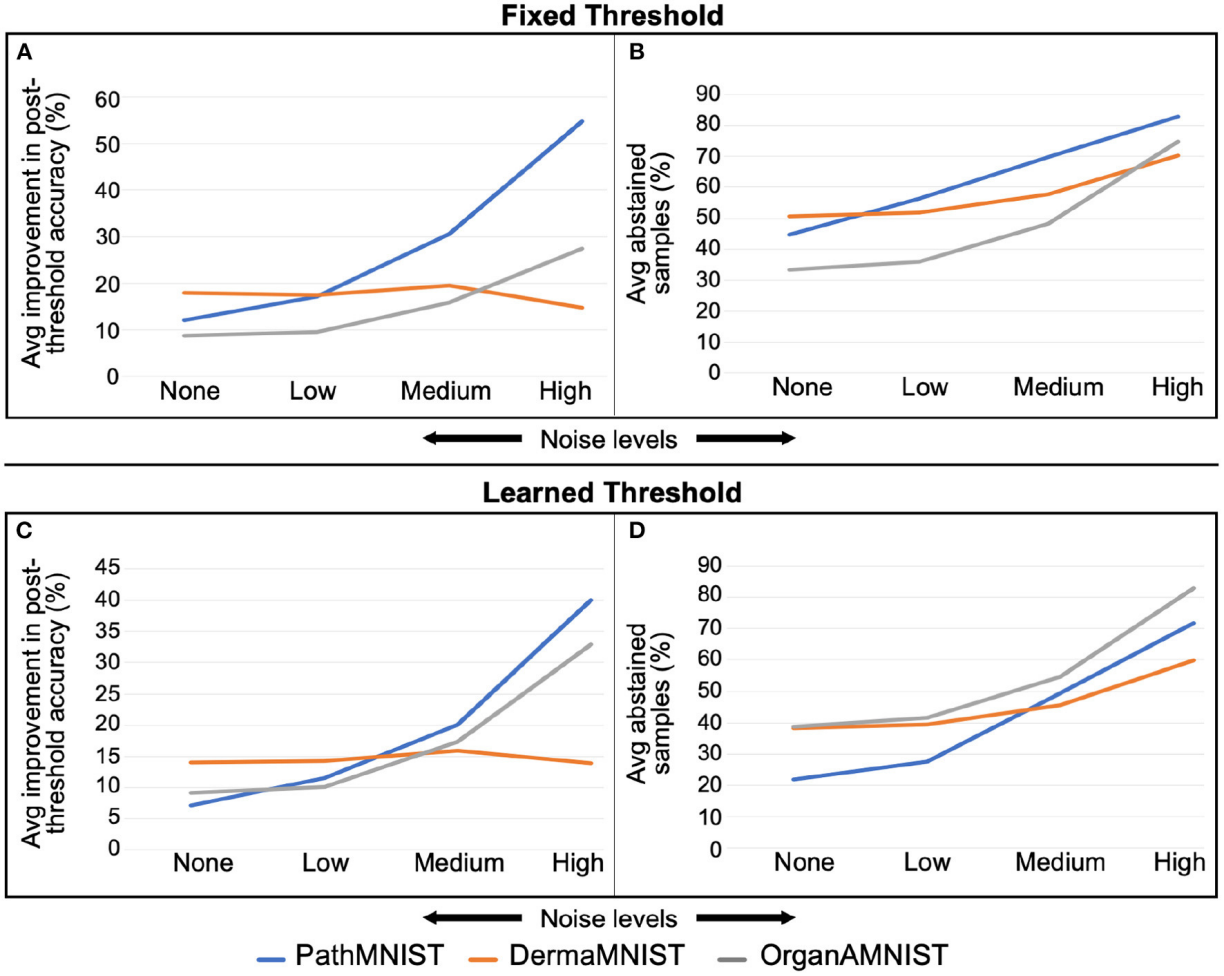
**(A,B)** are for fixed threshold. **(C,D)** are for learned threshold. Increase in average improvement in post-threshold accuracy and average percent abstained samples with rising Gaussian noise levels is visible. This trend varies depending on the dataset.

Figure 7 shows the comparison of accuracy improvement with noise level for fixed and learned threshold mechanisms. We observe that with the increasing noise levels, the average improvement in the accuracy is also increasing. However, the number of abstained samples is going high. The trend varies slightly based on the dataset.

### 4.6. Failure Detection Under Adversarial Attacks

#### 4.6.1. Fixed Threshold

Tables 8–10 present the performance of threshold methods under three different adversarial attacks. We observe that for the PathMNIST datasets (Table 8), the average increase in accuracy using the fixed thresholds is [11.7, 54.3, 15.2%] and the number of abstained decisions is [54, 58.7, 69.1%] for [“CW,” “PGD,” “FGSM”] attacks, respectively. For DermaMNIST (Table 9), the average increase in accuracy is [11.9, 27.9, 10.8%] and the number of abstained decisions is [54.5, 51.2, 54.3%]. Table 10 shows that for the OrganAMNIST dataset, the average increase in accuracy is [12.2, 30.1, 12.5%] and the number of abstained decisions is [50.2, 34.3, 51.1%] for [“CW,” “PGD,” “FGSM”] attacks, respectively.

The performance of the fixed threshold method under CW attacks is better than FGSM and PGD attacks, thus giving a higher increase in the post-threshold accuracy with fewer abstained samples for all three datasets. The performance of the fixed threshold method under PGD attacks is comparable to that under FGSM attacks. Among three tested datasets, the fixed threshold method performs the best under CW attack on OrganAMNIST, improving the average post-threshold accuracy to 99.4%. We also note that the CW attack completely fails the deterministic neural network with the test accuracy of 0.25, 0.25, and 1.00% for PathMNIST, DermaMNIST, and OrganAMNIST, respectively. On the other hand, the accuracy values for the BDNNs are 43.75, 64.25, and 69.25% for PathMNIST, DermaMNIST, and OrganAMNIST datasets, respectively. Compared to the deterministic neural networks, the BDNNs are inherently robust against attacks. By employing the fixed or learned threshold methods, BDNNs can detect the model failure and improve accuracy by abstaining from uncertain decisions.

The fixed threshold for FGSM attack shows that, on average, the percentage of abstained samples at the lowest level of FGSM attack is 45.2 and 51.3% for the PathMNIST and DermaMNIST datasets, respectively, which is less than the remaining three levels of the attack. However, the average accuracy improvement is relatively high, 15.4 and 17.5%, respectively. The average accuracy improvement is relatively low at higher attack levels, 9.5 and 3.0% for PathMNIST and DermaMNIST, respectively. However, the average abstained samples are relatively higher, 53.9 and 59.3%. Such patterns are observed when the model's accuracy drops considerably due to increasing noise/attack levels, and a strong overlap between the distribution of predictive variance for correct and incorrect samples exists. For OrganAMNIST, the highest average accuracy improvement under the FGSM attack is 17.1%, corresponding to the second level attack. The abstained samples are comparable to the lowest and third attack levels, 49.8% on average. The number of abstained samples is relatively high at the highest FGSM attack level, 64.2%; however, the average accuracy improvement is relatively low, 6.3%.

#### 4.6.2. Learned Threshold

The average accuracy improvement for the PathMNIST (Table 8) using the learned thresholds is [3.8, 43.9, −2.0%] and the number of abstained decisions are [35, 50.4, 40.3%] for [“CW,” “PGD,” “FGSM”] attacks, respectively. Table 9 for DermaMNIST shows an average increase in accuracy as [6.7, 21.1, 3.8%] and the number of abstained decisions as [45.3, 39.8, 46.0%]. Finally, for the OrganAMNIST (Table 10), the average increase in accuracy is [16.9, 30.6, 19.8%] and the number of abstained decision is [60.47, 40.9, 64.3%], for [“CW,” “PGD,” “FGSM”] attacks, respectively.

We note that the learned threshold method performed better under CW attacks than FGSM and PGD attacks. Consequently, this results in a higher increase in post-threshold accuracy with fewer abstained samples for all three datasets. Among all three datasets, the learned threshold method performed the best on the OrganAMNIST dataset under CW, and the lowest level of FGSM attack considering the accuracy improvement and percent abstained samples. The post-threshold accuracy is 99.8 and 99.6% for the CW and FGSM attacks, respectively. The learned threshold performs well for OrganAMNIST under all levels of FGSM attack (with a minimum accuracy improvement of 10% at the highest attack level with 74% abstained samples) compared to PathMNIST and DermaMNIST. For DermaMNIST, the method performs well under the lowest level of FGSM attack, improving average accuracy by 12% with the abstained samples of 39.4%. The learned threshold does not perform well on the PathMNIST dataset under PGD attack, reducing the post-threshold accuracy by 2.0%.

The performance of both failure detection methods, which, in turn, estimate a threshold value on the predictive variance, depends on the difference in the median variance values of correct and incorrect decisions. The performance of the proposed methods also depends on the overlap between the distributions of predictive variance values corresponding to correct and incorrect decisions. The performance of both methods will improve as the difference between the median predictive variance values of correct and incorrect decisions increases and the overlap between the two distributions reduces. These conditions vary based on the dataset used and the type of noise. The accuracy improved, and the number of abstained samples has a trade off based on the dataset. The user can choose a high accuracy improvement depending on the application and compromise on the high number of abstained samples and vice versa. The selection of the appropriate threshold method, learned or fixed, also depends on the application and the dataset. The proposed methods offer two main advantages, (1) generally, the post-threshold accuracy improves even at high noise levels or under adversarial attacks, and (2) the increase in the number of abstained samples and the increase in predictive variance indicates model failure.

## 5. Conclusion and Future Work

Self-assessment and failure detection requires accurate uncertainty estimation. However, the softmax output values produced by DNNs are overconfident and unsuitable for failure detection. We have used the recently proposed BDNN models that employ Bayesian methodology and simultaneously output the decision and uncertainty. This reliable uncertainty estimate forms the foundation of our proposed methods for detecting performance degradation and failure in DNNs. We have proposed two failure detection methods, fixed and learned threshold. The fixed threshold is defined by looking at the behavior of the predictive variance under changing noise levels (SNR). The second method uses a neural network to learn the threshold. Both methods filter out under-confident decisions (as “no decision”) having variance above the threshold level. This filtering out mechanism results in an increase in the accuracy of the remaining confident decisions. We test these failure detection methods on three medical imaging datasets, PathMNIST, DermaMNIST, and OrganMNIST. The results show a trade-off between the average improvement in post-threshold accuracy and the percentage of abstained samples. As the threshold rises, the number of abstained samples decreases, and the improvement in accuracy also decreases (keeping the noise level constant). As the noise level rises, the model becomes uncertain and its variance values rise. Additionally, the model accuracy drops under increasing noise levels, and the model is uncertain about its incorrect decisions. Rising noise level thus causes the number of abstained samples to increase (at the same threshold level), and the improvement in accuracy (post-threshold) also increases. The proposed method reflects performance degradation and model failure when the number of abstained samples increases and there is a rise in predictive variance.

There are some limitations of our study, which open avenues for future work. The first avenue is extending the proposed mechanisms to other datasets and model architectures. BDNNs, which are the base of our proposed failure detection methods, have been applied to other model architectures and can be extended to any architecture, including, Transformer, RNN and its variants (LSTMs/GRUs), and variants of CNN-based architectures. Since this mechanism is independent of the dataset, it can be extended to any medical dataset of interest. Furthermore, the proposed mechanism's performance depends on the accuracy and reliability of the uncertainty estimation. These failure detection mechanisms can be applied to other well-calibrated uncertainty techniques that quantify model confidence. In addition, for the proposed learned threshold mechanism, the model architecture and the loss function can be designed to enhance the performance for detecting failure with lower abstained samples giving a greater improvement in accuracy. We believe that our work will assist the broader acceptability of DNN's deployment for healthcare applications as a trustworthy and reliable solution.

## Data Availability Statement

The original contributions presented in the study are included in the article/supplementary material, further inquiries can be directed to the corresponding author.

## Author Contributions

GR and NB have equal contribution in conceiving the concept. DD carried out the initial mathematical derivation for the VDP. SA and SH devised the mechanism for failure detection. SA implemented the current code, carried out simulations, and wrote the manuscript. SA and GR verified the simulations, results, and proof read the manuscript. All authors approved the manuscript.

## Funding

This work was partly supported by the National Science Foundation Awards ECCS-1903466, OAC-2008690, and CRII-2153413. We are also grateful to UK EPSRC support through EP/T013265/1 project NSF-EPSRC: ShiRAS. Towards Safe and Reliable Autonomy in Sensor Driven Systems.

## Conflict of Interest

SH was employed by AMD Inc. The remaining authors declare that the research was conducted in the absence of any commercial or financial relationships that could be construed as a potential conflict of interest.

## Publisher's Note

All claims expressed in this article are solely those of the authors and do not necessarily represent those of their affiliated organizations, or those of the publisher, the editors and the reviewers. Any product that may be evaluated in this article, or claim that may be made by its manufacturer, is not guaranteed or endorsed by the publisher.
